# Developing, delivering, and evaluating an online course on socially assistive robots in culturally competent and compassionate healthcare: A sequential multiphase, mixed-method study

**DOI:** 10.1177/20552076241271792

**Published:** 2024-10-29

**Authors:** Irena Papadopoulos, Runa Lazzarino

**Affiliations:** 1 School of Health and Education, 4907 Middlesex University, London, UK; 2 Nuffield Department of Primary Care Health Sciences, 6396 University of Oxford, Oxford, UK

**Keywords:** AI transformation, health and social care workforce, AI socially assistive robots, digital health intervention, education and training, Massive Open Online Course, mixed-method, international study, cultural competence and compassion, care ethics

## Abstract

**Objective:**

Artificially intelligent socially assistive robots are a growing technology. There is no evidence-based, theory-informed, open access training targeting health and social care professionals on this advanced technology. This collaborative, international European project – the IENE 10 study – developed, delivered, and evaluated the first Massive Open Online Course on socially assistive robots.

**Methods:**

A sequential mixed-method design with five phases: (1) literature review; (2) development of the Transcultural Robotic Nursing curriculum model from the care ethics principles of cultural competence and compassion; (3) development of modules, learning units, and assessments; (4) choice of the digital platform, e-facilitators’ training, and definition of the evaluation strategy; (5) recruitment campaign. The methodology was collaborative among the six European partner institutions, who all contributed to each phase, from planning to the outputs. All project outputs and MOOC contents were translated into the four languages of the partners.

**Results:**

Training needs identified included: knowledge about social robots’ functionality; how to operate them; legal, ethical, and human rights' issues. The course had four modules: Awareness, Knowledge, Sensitivity and Competence, with four learning units each. E-learners (*n* = 240) were mostly based in the project partners’ countries and with no previous training on social robots. Graduated e-learners (*n* = 185) found their knowledge and skills enhanced, both in relation to social robots and cultural competence. The learning units and the overall quality of the course were rated between good and excellent.

**Conclusions:**

The IENE 10 project pioneeringly addressed the training needs of health and social care professionals in the era of AI social robots. The collaborative and sequentially phased design proved useful in the integration of a care ethics model. This work reflects the holistic approach needed for preparing professionals for the complexities of contemporary healthcare.

## Introduction

The rapid adoption of artificially intelligent (AI) technologies has transformed all industries globally, with the healthcare sector being no exception.^1–3^ Since 2017, the integration of AI technologies in healthcare has more than doubled, encompassing diverse applications such as diagnosis, patient engagement, clinical decision-making, and administrative tasks.^2,4,5^ Among the emerging trends, socially assistive robots (SARs) have garnered attention, particularly in elderly care and hospitals, gaining even more significance in the wake of the pandemic.^6–8^ A recent global review found that the most common settings of SARs deployment are hospitals and medical institutes, elderly care centres, occupational health centres, and private homes – with the top two countries of implementation being the USA and France.^1^

SARs, in essence, are designed to assist users with social interaction.^9^ They belong to those technologies devised to help older people enjoy a higher quality of life, by addressing some of the challenges faced by the ageing population.^10,11^ These robots are created to engender beneficial effects by supporting patients to express their feelings,^12^ provide comfort,^13^ alleviate anxiety^14^ and depression,^15^ and reduce loneliness.^16^ Accordingly, the most important functions of SARs identified are entertainment, educational entertainment, companionship, remote presence, and affective and physiological therapy.^1,6,17,18^ Enablers for their use have been shown to be enjoyment, usability, personalisation and familiarisation.^19^ However, attitudes towards SARs are still found to be ambivalent among the population,^20^ and they vary within the health and social care workforce too.^21^

Despite the advancements in SARs development, their successful implementation encounters obstacles similar to other technologies, including AI devices.^2^ Many factors can hamper an AI intervention uptake in health and social care practice, notably spanning from ethical concerns^3,22^ to culturally informed attitudes.^23^ Research investigating barriers to implementation is not abundant. While healthcare professionals’ attitudes were found to be generally positive towards SARs, nonetheless, technical problems, previous experience with technology, robots’ limited capabilities and negative preconceptions towards the use of robots were some of the barriers identified.^24^ Some studies highlighted the importance of setting social and legal boundaries for robots to ensure safety and privacy for the caregivers and the patients.^25–27^ Other concerns found regarded personal data loss or hacking and the reduction of ‘human touch’ in the profession.^28^ The attitudes of health and social care professionals are crucial, and they are influenced by professionals’ awareness and knowledge of social robots.^29^ Bridging the gap between technological innovation and professionals’ readiness requires addressing these concerns and providing adequate training and education.

The importance of ongoing training and education in relation to technological interventions in health and social care seems to hold exceptionally true for AI devices.^30–32^ The field of the literacy in AI technologies of the health and social care workforce is fast expanding^31,33,34^ – at least in high- and middle-income countries, with growing governmental and intergovernmental research, guidance and investments.^35^ However, training efforts related to SARs have been limited, revealing inadequacies in the preparedness of health and social care professionals.^36^ Research indicates that professionals desire to feel in control over SARs, ranging from basic operational skills to in-depth knowledge of ethical and legal aspects, data protection and privacy.^8,37,38^

This interventional study responds to the need for preparedness of the health and social care workforce towards the introduction of SARs in the sector. A Massive Open Online Course (MOOC) was developed, delivered, and evaluated as part of the 10th project of the Intercultural Education for Nurses in Europe (IENE) programme, IENE 10. MOOCs are web-based, open access courses that aim at unlimited participation. MOOCs are normally interactive, with user forums or social media discussions, and provide immediate feedback to quick quizzes and assignments.^39^ All the IENE studies are rooted in the model of Culturally Competent and Compassionate Care,^40,41^ which is an ethics of care and simultaneously an ethics of research and of teaching and learning (https://ieneproject.eu/training-models.php). The IENE 10 project ‘Preparing health and social care workers to work with socially assistive AI robots in health and social care environments’ was funded by the EU Erasmus+ programme. Five European countries with six partner institutions participated in the project: UK (with one of the two UK universities, Middlesex University, being the coordinator), Italy, Austria, Romania, and Cyprus.

## Materials and methods

**Figure 1.**
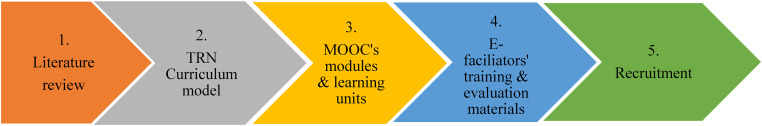
The sequential, multiphase mixed methodology of the IENE 10 study.

This study adopted a collaborative, sequential multiphase, mixed-method design to develop, deliver, and evaluate a MOOC to prepare health and social care professionals to work with AI SARs (Figure 1). The MOOC intended learning outcomes in relation to AI SARs implementation included the ability to: illustrate the practical aspects; evaluate both the negative and positive implications, including matters relating to inequalities and social inclusion; elaborate on relevant ethical issues. The project's activities started in September 2020 and concluded in February 2023.

Each project partner took the lead in one of the following five phases; however, the overall methodology was highly collaborative, in the sense that all partners’ teams contributed to all phases, discussing, reviewing, and approving all the steps within each phase, from planning to output. All the project outputs are publicly available on the IENE 10 project website (https://ieneproject.eu/IENE10/about-project.php) and the Erasmus+ Project Results Platform (https://erasmus-plus.ec.europa.eu/projects/search/details/2020-1-UK01-KA202-078802). The study intellectual outputs are numbered according to the study phase, dated, and the authorship belongs to the IENE 10 team, who is also the copyright holder in this study. All partner institutions involved in the project hold equal copyright and authorship rights on all the project intellectual outputs, regardless the fact that the leading teams appear on the outputs’ cover pages.

### Phase 1. Literature review

A literature review was undertaken between October 2020 and January 2021 to map the evidence on the views, attitudes, and training needs of health and social care professionals in relation to SARs. Searches were conducted at the international, European and national levels. National level searches were performed by each partner in their language. Inspired by the framework for scoping reviews by Arksey and O’Malley,^42^ the review was conducted using a combination of terms from the following three domains: attitudes/views/needs/perceptions; nurses/health and social care professionals/workers/midwives; and robots/robotics/social robots/socially assistive robots/AI robots. For scientific peer-reviewed literature, the following databases were searched: (a) EBSCO (CINAHL, MEDLINE, PubMed); (b) Google scholar; (c) Cochrane; (d) PsycINFO; (e) ScienceDirect; (f) Embase; (g) Web of Science; (h) IEEE Xplore digital library. For the grey literature, the search terms were used in the web search engine Google and included international, European, and national government reports, policy statements/papers, theses, project/white papers and evaluations, conference proceedings/bulletins/fact sheets, also as website contents. Regarding the scientific literature, only sources dated between January 2018 and January 2021 were included because previous literature reviews of the research team covering evidence up until 2018 indicated the absence of any learning and training programmes for health and social care workers in relation to SARs.^24,43^ For the grey literature, evidence sources from 2010 to January 2021 were instead considered, as a decade was deemed consistent with the development of this advanced technology. No review management software was used; each partner team employed *Word* and/or *Excel* programme to document their searches, sources, selection, and data extraction regarding views, attitudes, and training needs of health and social care professionals in relation to SARs. One reviewer in each partner team performed the searches and extracted the data. The selection of the eligible evidence was performed in tandem with a second reviewer in each team; divergences in relation to the inclusion of a source were resolved by team discussion. For those included sources in the project partners’ languages (Greek, German, Italian and Romanian), partners translated extracted data in English.

Following Peters and colleagues,^44^ presentation of the results mapped out the reviewed material in a tabular form and in a descriptive format that aligns specifically with the objective and scope of the review (Intellectual Output 1.1, March 2021). Twelve standardised tabulations of the results were created for the international and European literature, and for each set of results of the searches at the national level. Tables for the grey and scientific literature's results were kept separate. Subsequently, we analysed the results captured in the tables and in the descriptive synthesis adopting a theory-driven approach based on the Papadopoulos Model for Culturally Competent and Compassionate Care to identify themes.^45^ The process of analysis entailed a familiarisation with the data, followed by an extrapolation of key concepts and topics to identify patterns leading to the establishment of themes. Two team members independently conducted the analysis and had frequent meetings to reach consensus, until thematic saturation was reached, and final themes defined. In Intellectual Output 1.2 (May 2021), a description of the literature review's themes and sub-themes is provided, and themes were also captured with a visual diagram (Figure 4).

### Phase 2. Transcultural Robotics Nursing (TRN) curriculum model

Reflecting the consolidated methodology of the IENE programme, each IENE project is rooted in an ad-hoc model which encapsulates both the health and social care ethos and the educational one. To this end, three elements were set in dialogue to develop what was named the IENE 10 Transcultural Robotics Nursing (TRN) curriculum model: (a) the original Papadopoulos, Tilki and Taylor model of cultural competence in nursing,^41^ together with the more recently developed Papadopoulos Model for Culturally Competent and Compassionate Care.^40^ This element provided a logical, simple and flexile structure based on the model's four constructs of Awareness, Knowledge, Sensitivity and Competence, as well as the theoretical and ethical groundings of culturally competent and compassionate care; (b) thematic results of the literature review of Phase 1; (c) pre-existing knowledge and expertise of the IENE 10 team. These latter two elements (b) and (c) informed the development of contents within each of the four constructs of element (a) to create the TRN curriculum model. The development of the TRN model occurred between February and May 2021 (Intellectual Output 1.2, May 2021).

### Phase 3. MOOC's modules, learning units, and learning and assessment activities

Following Phases 1 and 2, the IENE 10 team developed the MOOC's structure and contents. Based on the TRN model, the MOOC was structured into four modules. All four modules were developed following a pre-defined structure, comprising of: aims, learning outcomes and four learning units (LUs), each focusing on a specific topic and offering further readings and resources, often with hyperlinks (Intellectual Output 3, December 2021).

**Figure 2.**
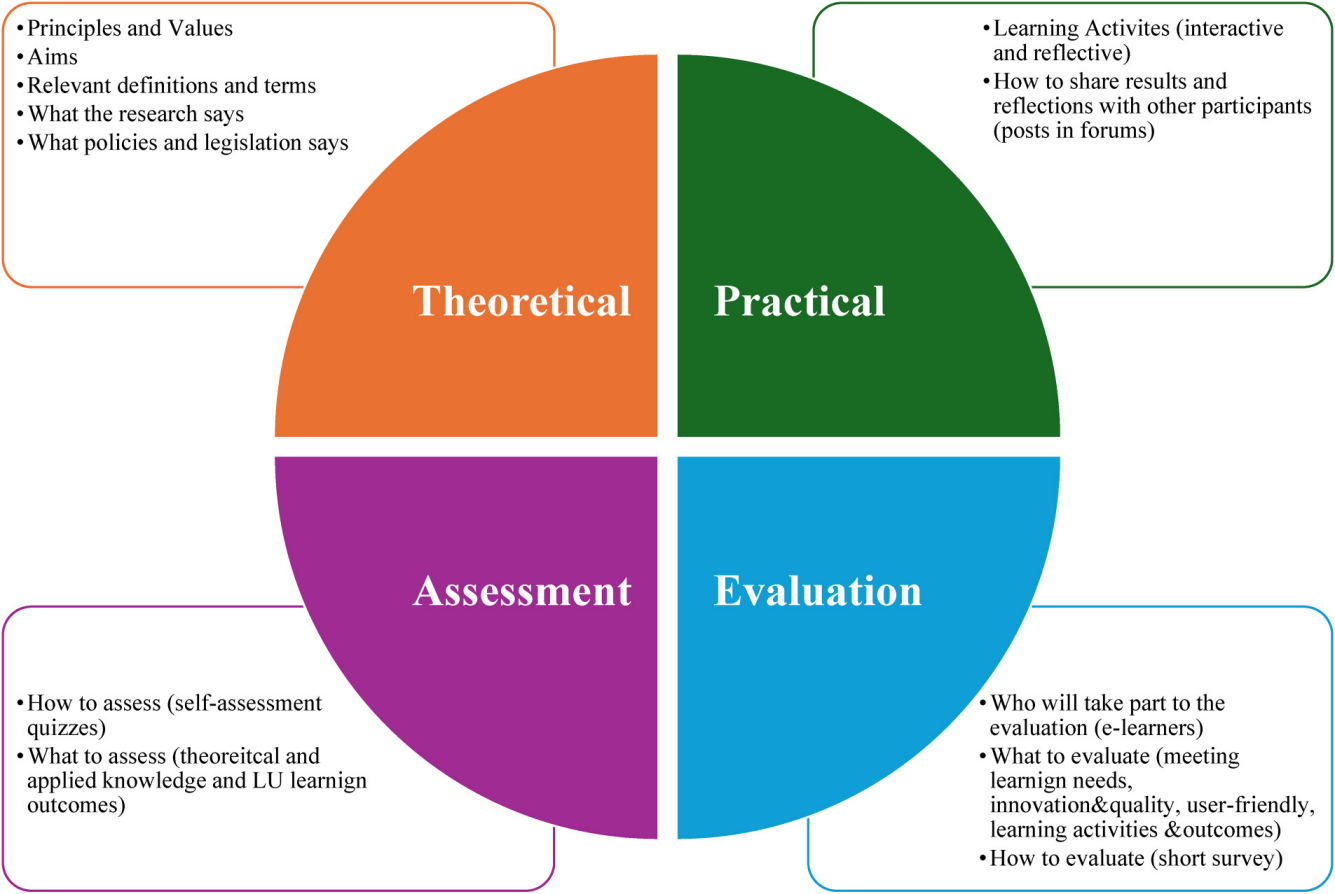
The four components of the IENE 10 MOOC learning units.

Each LU (*n* = 16 in total) was developed following a template refined and approved by the IENE 10 research team and which included theoretical, practical, assessment and evaluation components (Figure 2). Each project partner developed two or three LUs. The assessment of the participants’ performance (see below in the section ‘E-learners assessment and their evaluation of the MOOC’) was going to be based on the results of LU's quizzes and on a final evaluation task, together with their participation in the virtual class (e.g. posting comments into discussion boards and chats, including reflections on their MOOC experience and their ideas on how to apply the new knowledge and skills to their workplace, as well as on their future training needs). The assessment in the form of a self-assessment quiz was intended to focus on information contained in the LU and on the learning activities in that module. The whole MOOC's approved contents were going to be translated from English into the other four languages of the project partners.

### Phase 4. Pilot the MOOC, train the e-facilitators and approve the MOOC evaluation

During an in-person meeting of the IENE 10 team (Cyprus, October 2021), partners discussed a few platforms’ options used in the previous three IENE projects’ MOOCs, such as *Blackboard Learn* in tandem with *Slack*. The Austrian team proposed adopting the Austrian platform *iMooX* (https://imoox.at/mooc/?lang=en). Based on accessibility and easiness-to-use, budget considerations, ethical compliance, as well as on previous experiences with other platforms, consensual decision was taken to adopt *iMooX*.

After the MOOC contents were uploaded onto the platform, an in-person, 2-day meeting was held (London, September 2022) with three objectives: (a) train the MOOC e-facilitators, from the six partner institutions (*n* = 22); (b) discuss and approve the MOOC evaluation strategy and material; (c) pilot the MOOC content and technology with all the teams’ members and the recruited e-facilitators of the six partner institutions:

- The training of the e-facilitators included the educational model of the IENE studies and the specific digital features of the IENE 10 MOOC on iMooX. The training was led by the coordinating partner, the Middlesex University team. A facilitators training handbook had been produced with detailed guidance on, for example, the learning outcomes of the MOOC, the digital platform, tasks and responsibilities of the e-facilitators, the language policy and the MOOC timetable (Intellectual Output 6.1, June 2022). Tasks and responsibilities of the MOOC e-facilitators included: to monitor and encourage participation throughout the course; to answer questions on learning activities, materials and assessments relating to the course within 48 hours; to track participants’ progress, assess performance and assignments, and give encouragement and feedback. The e-facilitators were in charge of assigning the different achievement badges to reward participants and to provide the final attendance certificate.
- The IENE 10 MOOC evaluation strategy and materials were presented, reviewed, discussed and approved by the six partner institutions’ members (*n* = 24) to further ensure their effectiveness. The MOOC pre-course questionnaire collected basic demographic information, such as country of origin, professional qualification, occupation, self-rating digital skills and level of proficiency in the English language. The questionnaire's focus was on self-assessed learning needs of the participants in terms of their knowledge, skills, attitudes and their expectations in relation to the online course, with three free-text questions (Supplemental material 1). The post-course evaluation had two separate questionnaires: the first one asked participants to choose among two given lists of items, all those that applied, in relation to: (1) their perceived most important improvements in relation to knowledge, skills and understandings, and (2) their perceived impact of the course on different aspects, such as professional motivation and satisfaction, and language, digital and transcultural health competences. The second questionnaire wanted to explore how the course met the training needs (list of items) and the overall rating of the course (multiple choice, from ‘poor’ to ‘excellent) (Supplemental material 2).
  For each LU, a short survey was created using the SurveyMonkey app (https://www.surveymonkey.com/). The criteria for the LU's evaluation were: coverage of the identified learning needs; innovation and quality of the content and training materials; intuitive and friendly presentation; relevance of learning activities; efficiency for achieving established learning outcomes.
  The pre- and post-course evaluation questionnaires, as well as the LUs evaluation surveys, were designed to align with the core objectives outlined in the guidelines of the project funder, the EU Erasmus + Programme.^46^ While the questionnaires were not formally validated in a traditional sense, their content and structure were rigorously developed to reflect the requirements of the project evaluation strategy.^46^ This approach ensured that they were robust and tailored to effectively assess the specific outcomes of the IENE 10 MOOC. The items of the questionnaires were also derived from established metrics used in previous IENE projects,^47–49^ supported by the same funder, and were adapted to the unique context of this MOOC, ensuring relevance and applicability.
- The MOOC curriculum (Intellectual Output 3, December 2021), including the LUs and the assessment tools, had been internally reviewed by the IENE 10 team via email exchanges. During the second day of the in-person meeting, the whole MOOC material transferred onto the digital platform was pilot tested with the 24 project members of the IENE 10 team. The team held a daylong mock attendance to the MOOC, where each member, from their personal computers, logged into the MOOC platform, went through the registration questions, the introductory material and each of the four components of the16 LUs, including the quizzes. The team had planned to pilot the six weeks of the MOOC in 6 hours, and for each hour, 30 minutes were dedicated to individual work and 30 minutes to plenary group discussion where participants were expressing their questions, comments, doubts, issues encountered and suggestions for amendments. As a result of this pilot test, the MOOC registration questions were streamlined, and some of the titles of the discussion forums were changed for better clarity, as well as other terms and sentences. Technical aspects were discussed and, where possible, simplified to make the MOOC as widely accessible as possible. All these minor amendments received consensual approval.

One of the advantages of online courses is that the digital platforms – as also *iMooX* – allowed the collection and interrogation of data relative to the MOOC e-learners, including their attendance, participation during the course, and performance in the quizzes and the final assessment. The chosen platform could also perform simple descriptive analysis of such e-learners’ data as well as the generation of simple visual diagrams. Descriptive statistics of the pre- and post-MOOC questionnaires (Supplemental Materials 1 and 2), as well as of the short surveys for each LU, were instead conducted separately, with the support of *SurveyMonkey* and *Excel* programmes. All this wealth of data, accompanied by descriptive summaries, is offered in the IENE MOOC final evaluation report (Intellectual Output 6.2, December 2022).

### Phase 5. Recruitment strategy and e-learners

**Figure 3.**
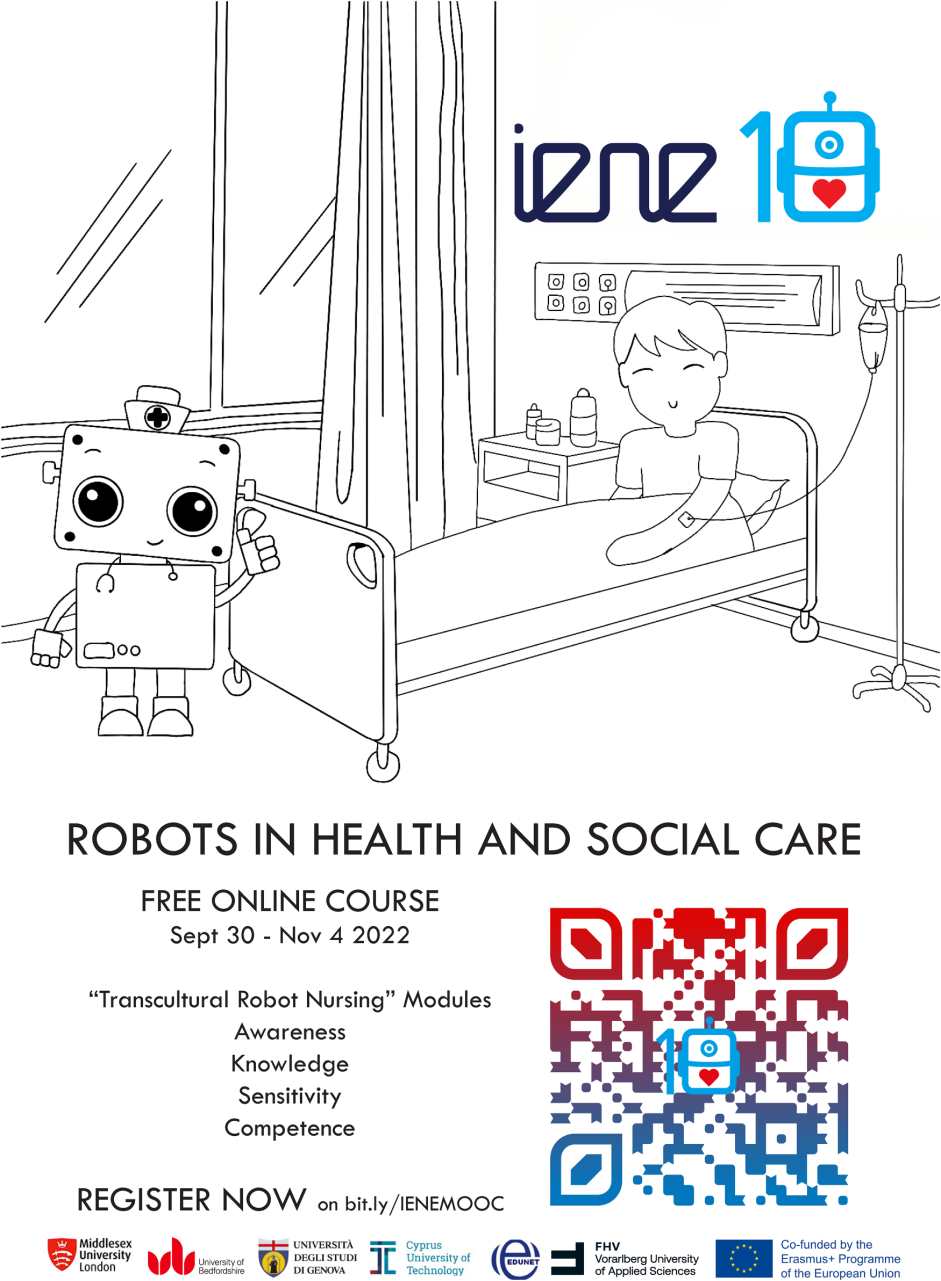
Example of IENE 10 MOOC recruitment material (Poster).

An articulated recruitment strategy was developed (Intellectual Output 5, June 2022). The strategy capitalised on the experience gained with previous IENE MOOCs. Recruitment of MOOC participants relied on nonprobability sampling based on convenience and snowballing strategies, to address the study objectives. Each project partner recruited at the national level, in their language, exploiting their professional networks and institutional channels. The following targeted groups were relevant for the IENE 10 study and needed a specific recruitment approach: educational and research network; associated companies and other partners (e.g., care homes, hospitals, mobile care services, rehabilitation centres, professional associations); universities and higher education institutions, starting from the partners’ ones where specific faculties and teaching modules were identified and targeted. The enrolment campaign was supported by an informative package made of a 30” promo videoclip, a short and a longer videoclip offering an overview of the MOOC, a presentation, a flyer, and a poster (Figure 3). All materials were translated in the languages of the partners’ countries, including the subtitles for the videos. Free enrolment in the MOOC was advertised online via email lists, institutional websites and emailing, local press and social media. The MOOC was also presented in person during university open days, meetings, scientific or informal conferences, formal and informal meetings with lecturers, partners, students and alumni, and national seminars (one in each partner institution).

The IENE 10 MOOC recruitment strategy targeted professionals, teachers, and students in the healthcare sector – especially in nursing and midwifery – and in social care and robotics, based in any countries around the world. Recruitment started in June 2022 and enrolment in the MOOC was allowed throughout the duration of the MOOC, up until 2 weeks prior to its closure.

### Ethics and rigour

This study required approval only in relation to the phase of the delivery of the MOOC, where personal data were collected from e-learners in the pre- and post-course questionnaires. These questionnaires collected minimal personal data (Supplemental materials 1 and 2). At pre-enrolment, e-learners were asked to read a detailed information page of the project and the terms and conditions of the MOOC. These included clarifications about the possibility of withdrawing at any time and that personal data would be anonymised. The Romanian partner institution, Edunet Association, was responsible for the evaluation of the course, and, to this end, for the data collection and analysis. To give their informed consent, e-learners were asked to click on the button ‘Enrol Now’.

Rigour and trustworthiness were ensured by the combination of methodologies and data sources, and the tight collaboration of an international, multi-disciplinary research team. This study design entailed a robust triangulation, covering the four main types of method, data source, investigator and theory triangulation.^50^ Frequent international and national team meetings, which due to COVID-19 were conducted more online than in person, constituted a relevant methodological tool towards the rigour of the study's highly collaborative design. Some team members were transcultural nursing scientists, with a long-term experience in culturally competent and compassionate nursing, others were specialised in education and training, others were social psychologists and others robotics engineers. The challenges experienced by the IENE 10 team primarily had to do with the number of translations, assuring their authenticity and accuracy. Each phase of the study methodology was described in an open access document named Intellectual Output, reviewed and published on the project website (https://ieneproject.eu/IENE10/about-project.php). As described above, quality of the MOOC's delivery was ensured by a face-to-face training of the e-facilitators, supported by a comprehensive handbook (Intellectual Output 6.1, June 2022). The MOOC was piloted with the facilitators and the large IENE 10 team, and the evaluation kit consisted in a comprehensive set of tools (Intellectual Output 6.2, December 2022).

## Results

### Literature review

**Figure 4.**
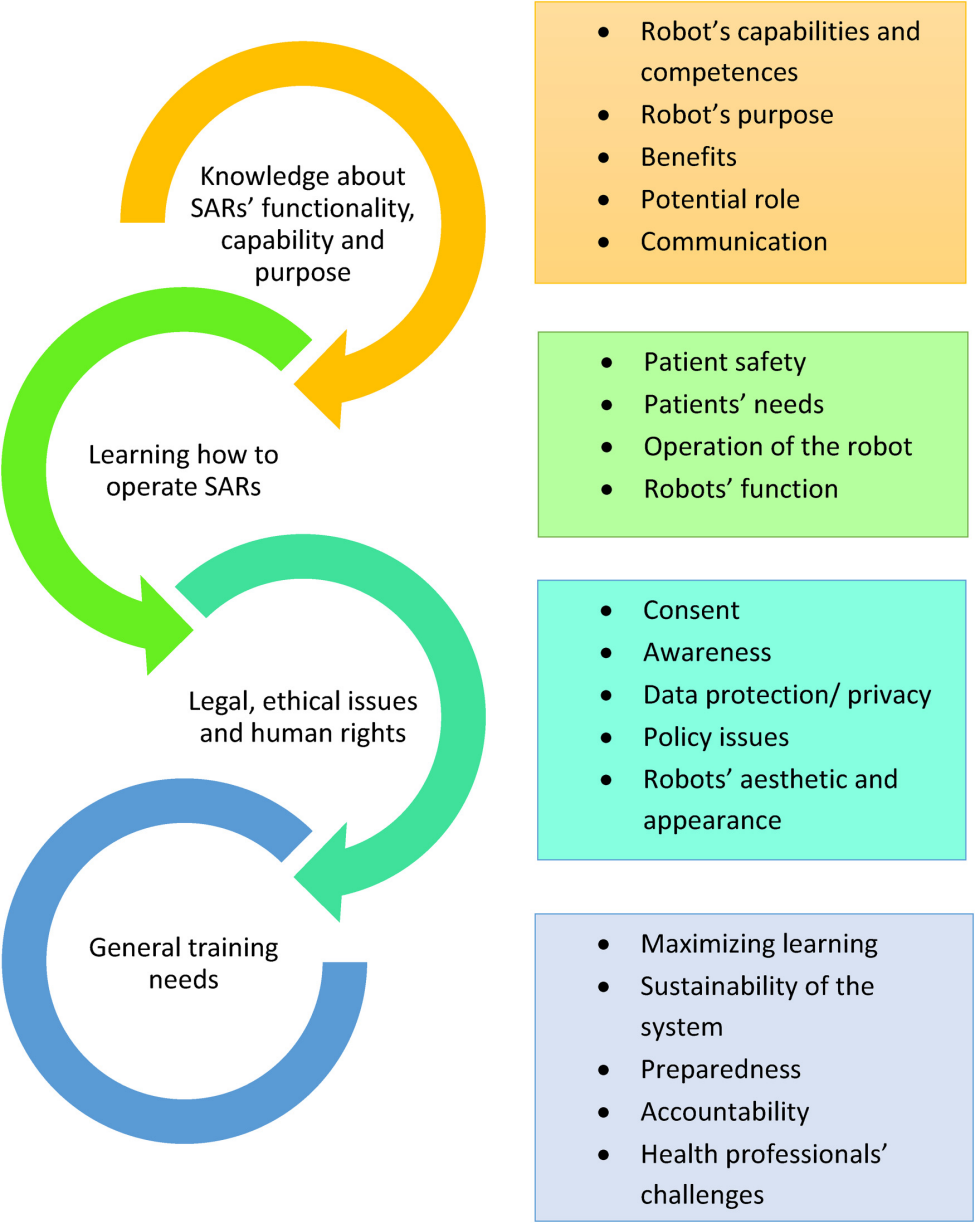
The four themes, with sub-themes, of the literature review.

The review of the literature yielded 25 international and European relevant sources, whereas 124 sources were included from the national level searches. The descriptive and tabular syntheses of the results are available from the IENE 10 project webpage (Intellectual Output 1.1, March 2021). Four macro-themes were identified: Theme 1. Knowledge about SARs’ functionality, capability and purpose; Theme 2. Learning how to operate SARs; Theme 3. Legal, ethical issues and human rights to consider when working with SARs; Theme 4. General training needs. Details of the sub-themes are offered in Figure 4 and a more thorough description is offered in the Intellectual Output 1.2 (May 2021).

### IENE 10 TRN curriculum model and MOOC's modules, learning units and activities

**Figure 5.**
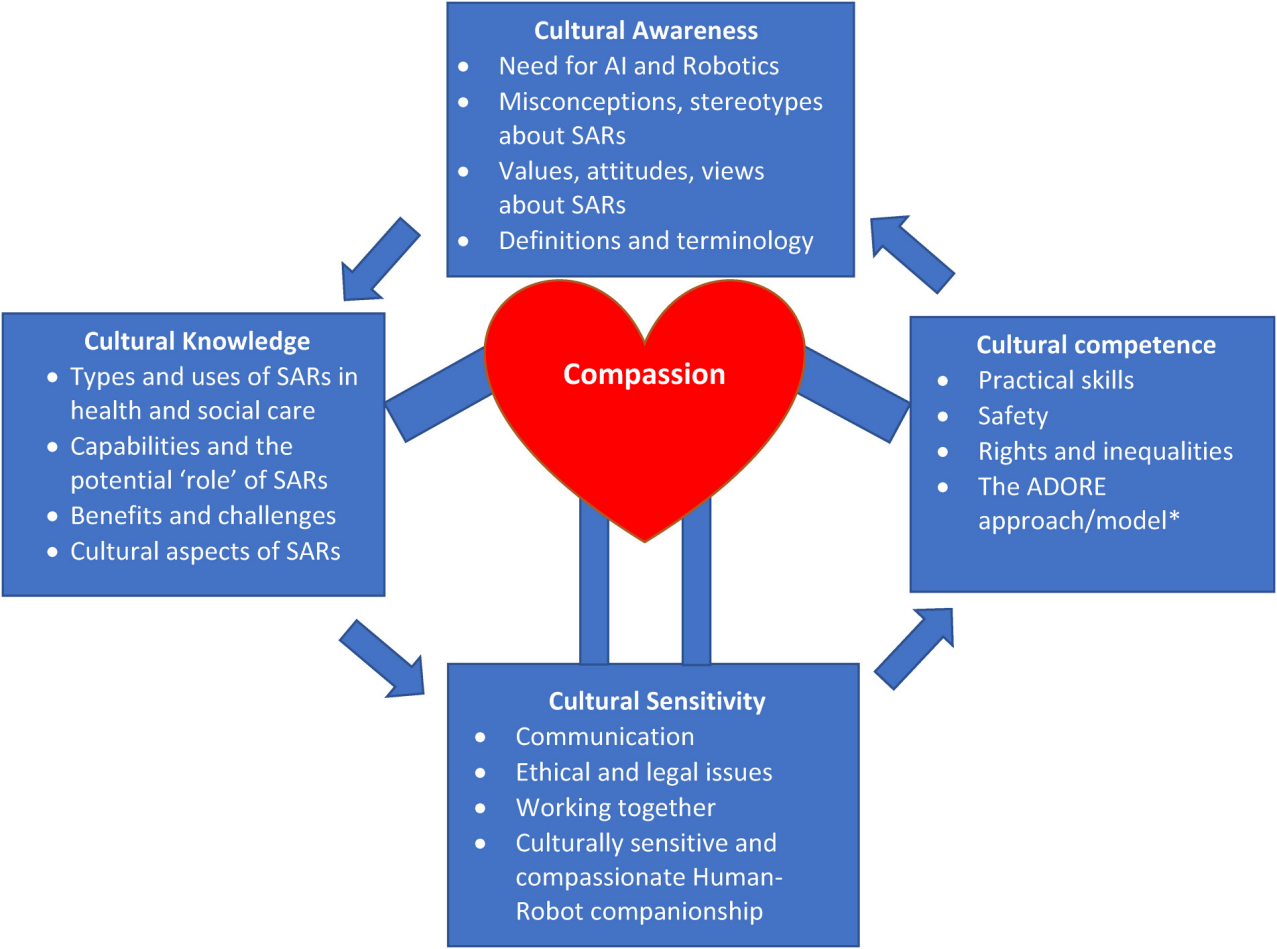
The IENE 10 Transcultural Robotics Nursing (TRN) curriculum model.

The final TRN curriculum model was visually represented with a diagram comprising the four constructs of Awareness, Knowledge, Sensitivity, and Competence with sub-constructs rooted in culturally competent and compassionate care and adapted for the IENE 10 study (Figure 5).

The IENE 10 MOOC four modules were responding to the four constructs of the TRN curriculum model (Table 1). The first module focused on increasing the awareness relative to the need for and the use of SARs in health and social care. The second module dealt with cultural knowledge and aimed to familiarise participants with different types of SARs. The third module addressed the theme of cultural sensitivity in the use of SARs; and the fourth module focused on cultural competence and the implementation of culturally competent SARs. Details of each module and of the LUs’ topics are illustrated in Table 1.

**Table 1.** IENE 10 MOOC modules and learning units topics.

	Learning Unit 1	Learning Unit 2	Learning Unit 3	Learning 4
**Module 1. Awareness**	Definitions, terminology, abbreviations and course orientation	The needs for SARs: the professional and the patient's perspective	Misconceptions and stereotypes about robots	Cultural values, attitudes and views about SARs
**Module 2. Knowledge**	Types and uses of SARs in different health and social care settings	Capabilities and the potential ‘role’ of SARs: imaginary vs existing robots	SARs’ benefits and challenges in the context of health and social care	Cultural aspects in designing and interacting with SARs
**Module 3. Sensitivity**	Human–robot interaction and communication of personnel, family, carers and robots	Ethical and legal issues of SARs implementation	Working together: humans–robot collaboration	Culturally sensitive and compassionate human–robot companionship
**Module 4. Competence**	Practical skills and general abilities in the interface with robots and artificial agents	Safety in the use of robots for the care of the patient/client	Rights of patients/clients and inequalities in access to SARs	The ADORE approach/model^a^

^a^ **The ADORE model/approach.** Developed by I. Papadopoulos during the CARESSES project (2017–2020), the ADORE acronym stands for Assess, Do, Observe, Revise and Evaluate. The ADORE model/approach helps the robot to understand the cultural aspects and importance of the humans’ actions, processes and decisions, all of which are essential to transcultural robotic nursing. The robot can use ADORE steps to make its cultural assessment (A), act on it (D), observe the results (O), if needed revise its actions (R) and then evaluate the outcome (E) (Papadopoulos et al., 2022).

Each LUs had four components, following the agreed template (see above Methods, Phase 3). That template resulted in the following IENE 10 LUs’ structure (Figure 2): (1) The theoretical components contained: principles and values; aims; relevant definitions and terms; what the research says; and what policies and legislation say. (2) The practical components contained two/three learning activities, which would ask e-learners to read a text/watch a video/visit a webpage and answer some questions; or reflect on their learning/a given scenario and answer a few questions; or complete a riddle/crossword puzzle online; or play an online game, such as a card sorting challenge. As it is generally the case for MOOCs, also the IENE 10 MOOC was based on activities that were designed to enhance the participants’ knowledge and skills, but also to support interaction with the other peer e-learners. For this, as part of the learning activities’ tasks, e-learners were encouraged to communicate and share their reflections and experiences on both the MOOC's general forum, and the different forums created for each LUs, for language competence and for technical issues. (3) The assessment component consisted in quick quizzes, for example with true/false or multiple-choice answers, or where participants had to complete sentences, or rank concepts, or other online tests and games. In the assessment activities, e-learners would obtain a specific score, as the aim was to confirm what a participant had learned, demonstrating whether they had achieved the learning outcomes of the unit. The quizzes were scored automatically, and the participants were notified whether they passed or failed the assessment. If they failed, they could retake the quiz. (4) The evaluation component was a quick survey aimed to provide direct feedback to the IENE 10 team for further improvement of the content (see above Methods, study Phase 4).

To the importance of culture and compassion in health and social care was dedicated the fourth unit in each of the four modules (Table 1). The summative assessment consisted in the production of a reflective output capturing their MOOC learning journey and a plan of action on how their learning could be used and shared. The whole curriculum, including all the components of the 16 LUs, such as the learning activities, the quizzes, and the LUs evaluation surveys, is available as IENE 10 Intellectual Output 3 (December 2021) on the project website.

### IENE 10 MOOC e-learners and course delivery

**Figure 6.**
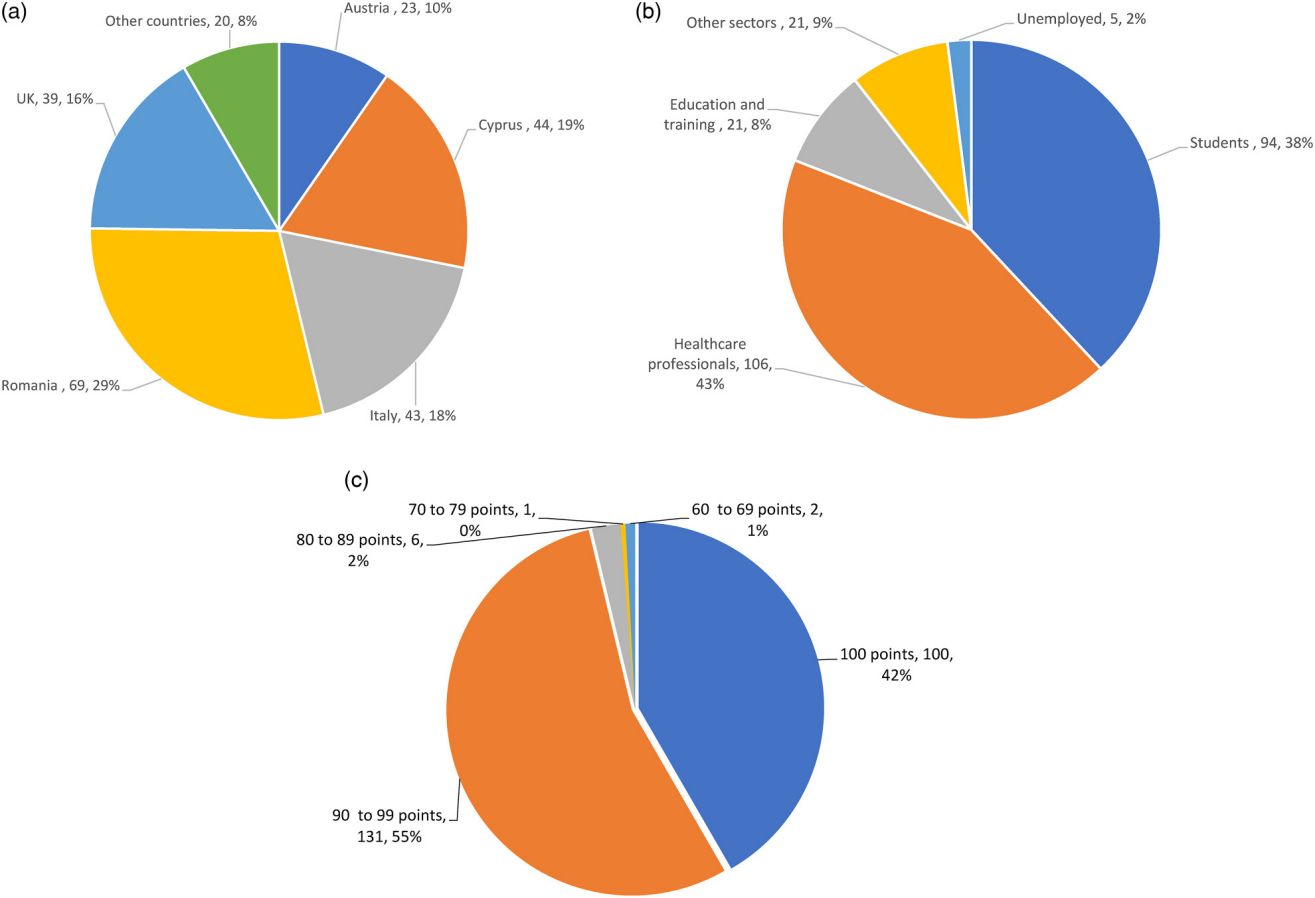
IENE 10 MOOC participants characteristics and scores: (a) MOOC participants’ country, (b) MOOC participants’ professional sector, (c)MOOC participants’ averages of the quizzes’ scores.

The MOOC was delivered over 6 weeks, between September and November 2022, comprising of an introduction and orientation week, 1 week per module, and a catch-up and evaluation week. A total of 450 e-learners enrolled in the MOOC, whereas 240 participants filled the pre-MOOC questionnaire (53%) – most of whom were based in the project partners’ countries, and 20 only (8%) in other countries worldwide (i.e., Philippines, Morocco, India, Colombia, Ireland, Hungary, China, The Czech Republic, Estonia, Spain, Germany, Portugal) (Figure 6(a)). Most participants were working or preparing to be professionals in health and social care (n = 48, 20%), the rest worked in robotics and engineering sciences and other specialties (Figure 6(b)). Only 27 (11%) participants declared that they previously attended a course on the topic of robots in health or social care and 57 (24%) that they had attended any type of MOOC.

### E-learners assessment and their evaluation of the MOOC

Data on: (a) e-learners performance assessment in the course and on (b) their evaluation of the MOOC are jointly reported in the IENE 10 MOOC evaluation report (Intellectual Output 6.2, December 2022).

- Assessment of e-learners. A total of 240 participants completed the LUs quizzes, out of which 205 (85%) completed all the 16 quizzes and 35 (15%) only some of them. E-learners’ averages of the quizzes’ scores are offered in Figure 6(c). The summative assessment was submitted by 191 (80%) e-learners. A total of 185 participants graduated and received a certificate of completion/attendance (77%). In addition, on every topic, there were engaged discussions with an average of 62 posts per forum. The highest number of posts were on the forums ‘Introduce myself’ (*n* = 323) and ‘Synthesis and Reflection for Final activities’ (*n* = 436).
- MOOC evaluation by the e-learners. The most selected expectations that e-learners expressed in pre-MOOC questionnaire (Supplemental material 1) included broadening and increasing their knowledge and awareness of SARs in relation to their capabilities and uses in the sector as well as to the advantages and disadvantages of their implementation at different levels of the profession – from career progression to personalised care. Other expectations regarded the enhancement of cultural, digital and language competences.

The e-learners (*n* = 196, 82%) who completed the MOOC post-course questionnaires (Supplemental material 2) declared that they had increased their professional knowledge and understandings of SARs’ uses, as well as of issues related to their introduction in health and social care, and had improved their level of professional skills (Table 2). Fifty-eight per cent of e-learners (*n* = 114) declared that they increased their language skills, and 60% (*n* = 118) that they increased their digital competence and skills for using different technologies. Additionally, 66% (*n* = 129) of respondents declared that they had improved their cultural knowledge and skills of culturally competent communication and 74% (*n* = 145) that they had acquired a greater understanding and responsiveness to social, ethnic, linguistic, and cultural diversity issues. The LUs were largely appreciated as good and very good (*n* = 192, 98%). The overall quality of the MOOC was rated excellent by all the 28 (14%) participants who completed it. In the Intellectual Output 6.2 (December 2022), more detailed evaluation results are offered, including quotes form the free-text answers of the MOOC pre-course questionnaire (Supplemental material 1).

**Table 2.** Results of the post-course evaluation questionnaire 1 for MOOC participants: the first 14 most selected items.

Item	Respondents (*N* = 196)	Percentage (%)
1. I have increased my knowledge on TRN specific issues in health and social care education and practice	**162**	87.57
2. Awareness of some of the main reasons for Socially Assistive Robots (SARs) being used in health and social care settings	**162**	87.57
3. Awareness of some of the main misconceptions and/or stereotypes that currently exist regarding the use of SARs in caring patients/clients	**142**	76.76
4. Knowledge of different types of SARs and their various uses in health and social care	**142**	76.76
5. Knowledge about the capabilities and the potential ‘role’ of transcultural SARs in health and social care	**140**	75.68
6. Knowledge about ethical and legal concerns associated with the safe implementation of SARs in health and social care	**140**	75.68
7. Knowledge of some of the benefits and challenges related to usage of transcultural SARs in health and social care	**138**	74.59
8. Awareness of the cultural values, attitudes and views that health and social professionals may have about SARs	**135**	72.97
9. Understanding of how transcultural SARs can provide culturally sensitive and compassionate human–robot companionship to patients/clients in health and social care settings	**133**	71.89
10. Understanding of the importance of transcultural communication between health and social care staff, the client and his/her family members, carers and SARs	**132**	71.35
11. Knowledge and understanding of potential issues related to physical and psychological safety of the patient/client when implementing SARs in health and social care	**131**	70.81
12. I have improved the levels of skills, linked my professional profile	**131**	70.81
13. Understanding the significance of collaborative teamwork between different stakeholders, including SARs and client/patient SARs and health/social care workers, towards ensuring quality of patient/client care	**129**	69.73
14. Understanding about the practical knowledge and skills needed to work with transcultural SARs in health and social care	**122**	65.95

## Discussion

The health and social care workforce needs to be exposed to opportunities for education and training in digital and advanced technologies, as these are inexorably becoming part of health and social care.^51–54^ The incorporation of AI devices and robotics into training programmes and frameworks is becoming mandatory given the fast-changing landscape of digital health.^51,55,56^ This study responds to such imperative call with a practice- and impact-oriented project advancing both the evidence base in the field and the preparedness of MOOC participants and of the IENE 10 research team. The positive outcomes of the IENE 10 MOOC should encourage health and social care organisations to prioritise the continuous professional development of their staff in the area of digital technologies, recognising the importance of keeping pace with technological advancements to provide high-quality, culturally competent care.

The literature review of this study highlighted the need for the health and social care workforce to be equipped with better knowledge about what SARs can do; how they can be used and be useful to professionals, patients and their family members. These findings are consistent with the growing, but currently limited implementation of SARs in the sector, whereby hands-on experience among the population is scarce, hence training should be broad and basic.^8,36^ The literature review's results suggested that preparedness in relation to ethics and human rights is also called for, as literature on training needs in digital literacy and AI robots in health and social care has amply shown.^3,55,57–59^

The collected data on the MOOC attendance, participation, assessments, and evaluation revealed improvements in participants’ knowledge levels after completing the MOOC. The IENE 10 MOOC managed to enhance e-learners’ understanding of practical aspects, benefits and challenges of SARs implementation, relevant ethical issues, and Equality, Diversity, and Inclusion (EDI) matters. The success of the IENE 10 MOOC in enhancing participants’ knowledge and cultural competence regarding SARs could serve as a model for the development of similar training programs in other emerging technologies within the health and social care sector, such as virtual reality, telemedicine or wearable devices.

The European Commission^60^ stated that AI technology is a strategic one that offers many benefits to citizens, businesses, and societies, provided that it is human-centred, ethical, sustainable, and that respects fundamental rights and values. Echoing these fundamental principles, this study advanced the fervent corpus of the ethics of nursing and AI.^57,61,62^ The ethics of care of this study is simultaneously an ethics of research and of teaching and learning, which is expressed in the model of Culturally Competent and Compassionate Care^40^ and has been adapted in this study into the TRN curriculum model. The model has guided different phases of the study – from data analysis and MOOC learning principles and structuring, up to the MOOC contents. The values of person-centred and humane care simultaneously speak to the dimensions of cultural specificity, transcultural competence, and universal human rights. Additionally, this applies to all the actors involved in care: professionals, patients, family members, and SARs themselves.^63^

The IENE 10 MOOC has been unique in both developing the first, open access training in this topic and to have it rooted in an applicative model of ethics of care. As mentioned, our results illuminated the transformative impact of the IENE 10 MOOC on participants’ cultural competence. Healthcare professionals reported enhanced awareness of cultural nuances, improved sensitivity in robot–human interactions, and a greater sense of competence in integrating SARs into their practice in a person-centred way. Some of the most selected impacts of the MOOC by e-learners included tangible improvements in their healthcare practices, with enhanced patient communication and culturally sensitive care delivery. The MOOC's real-world applicability was a testament to its alignment with the evolving needs of healthcare professionals. These results underscore the effectiveness of the TRN model in addressing the identified gaps and equipping professionals with the necessary awareness, knowledge, sensitivity and competence. Cultural competence is an essential approach to all services users, yet arguably even more crucial when preparing the introduction of AI SARs – around which there are persisting fears that care can be de-humanised^28^ and not accepted on the basis of cultural dissonance.^23^

The international and interdisciplinary design of this intervention increased participants’ capacity in transcultural and collaborative research. Transnational discussions facilitated the exchange of diverse perspectives and enriched the learning experience through the different MOOC's digital forums. The e-facilitators’ efforts to bridge cultural differences and create a supportive online environment was instrumental in the success of the MOOC. The teaching and learning model of the MOOC was indeed based on EDI principles. The course was not only fully accessible, but also rooted in inclusive educational values, such those of life-long learning, peer learning, the flipped-classroom, and self-paced, interactive and autonomous learning.^64–66^ The development of the IENE 10 MOOC has followed established principles of online learning engagement, from a behavioural to a cognitive and emotional one.^65,67,68^ MOOCs can be particularly effective in widening the catch of interested learners on a global scale, also in harder to reach context and among an overloaded workforce or studentship.^69^ Some of the challenges faced by participants, including varying technological literacy and cultural adaptation difficulties, can be useful to inform recommendations for future iterations, emphasising the need for tailored support mechanisms and expanded cultural competence components. Future research should concentrate on comparing the effectiveness of the IENE 10 MOOC with other training methods, such as in-person workshops or self-paced learning materials, to determine the most efficient and impactful approach to educating health and social care professionals about SARs and other emerging technologies. Additionally, longitudinal and follow-up research would be needed to assess the long-term impact of the MOOC on participants’ professional practices, patient outcomes and the integration of SARs in their respective health and social care settings. This could provide valuable insights into the sustainability and effectiveness of the knowledge and skills acquired through the course.

The IENE 10 study has addressed one of the most important interventions of our times, the deployment of AI SARs in health and social care. This innovation becomes even more pressing in the likelihood of recurrent infectious pandemics.^70,71^ The role of health and social care professionals is pivotal during emergencies and crises.^72^ If turned into a permanently available training tool, the IENE 10 MOOC could be used by social and healthcare professionals and volunteers, as an autodidactic and train-the-trainers instrument, to be better equipped towards the use of SARs at the next pandemic or disaster.^73^ These post-COVID insights were corroborated by the participants to the course who expressed that the IENE 10 MOOC was at the same time timely, innovative, and of direct practical use responding to their evolving training needs.

## Limitations

This study is not without limitations. Despite compensated by a triangulation of the multiple methodologies and researchers, standard limitations and biases of literature reviews are present^74^ – such as the lack of studies’ quality appraisal and the provision of a descriptive overview, rather than a synthesised result from specific questions. Additionally, some evidence sources were available in the partners’ languages and only a summary in English was produced: this limited the elaboration of a more nuanced narrative synthesis and subsequent analysis. Single partners, however, have been encouraged to work at their own research outputs in their languages.

Other important limitations concern the MOOC delivery in itself and participants’ recruitment, characteristics, and retention. Despite the thorough recruitment strategy, the risk of nonresponse bias remained high. It is known that online courses, such as MOOCs, can reach and be attended by a vast number of students, at their pace; however, access can be also precluded to groups without internet connection facilities and digital literacy. This MOOC was designed by a European research team and was mainly delivered to European or higher-income countries’ participants. More attention to cater for a more diverse audience of learners must be paid in future iterations and scaling-up, to ensure the optimal inclusivity of the IENE 10 or similar MOOCs.^75,76^ Additionally, a limited amount of sociodemographic data were collected in the MOOC pre- and post-course questionnaires, in line with the ethical principle of reducing the collection of personal data which are not strictly necessary to the study. This precludes an assessment of e-learners’ representativeness of the target population. The very general options given to participants for their qualification and occupation, such as ‘healthcare professional’, do not allow any conclusions to be drawn about specific healthcare professional roles, such as the participating nurses, midwives, or doctors. Additionally, the fact that no sociodemographic data were collected in one of the two post-course questionnaires hampers the interpretation of findings in relation to the e-learners’ profiles and job roles. The further limitation is the relatively high MOOC dropouts, where 41% of those who initially enrolled eventually graduated. Despite this being a known phenomenon due to a number of factors,^77^ better retention strategies should be implemented in future iterations. Finally, COVID-19 and key partners’ illnesses hampered the conduction of all the in-person meetings originally planned and caused some delays to the project timeline.

## Conclusion

The IENE 10 project, encapsulated in the MOOC, stands as a ground-breaking initiative in addressing the training needs of health and social care professionals in the era of SARs. The highly collaborative and sequentially phased design proved useful in the integration of a care ethics model, a carefully curated curriculum by an international research team, and a dynamic MOOC platform. This work reflects the holistic approach needed to preparing professionals for the complexities of contemporary healthcare.

As technology continues to advance, initiatives like the IENE 10 study serve as beacons, guiding professionals towards a future where the seamless integration of SARs aligns with cultural competence, ethical considerations, and a commitment to patient-centric care. The results and discussions emanating from the project pave the way for ongoing research for the development, implementation, and optimisation of tailored training programs that bridge the gap between emerging technologies and the readiness of health and social care professionals worldwide.

## Supplemental Material

sj-doc-1-dhj-10.1177_20552076241271792 - Supplemental material for Developing, delivering, and evaluating an online course on socially assistive robots in culturally competent and compassionate healthcare: A sequential multiphase, mixed-method study Supplemental material, sj-doc-1-dhj-10.1177_20552076241271792 for Developing, delivering, and evaluating an online course on socially assistive robots in culturally competent and compassionate healthcare: A sequential multiphase, mixed-method study by Irena Papadopoulos and Runa Lazzarino in DIGITAL HEALTH

sj-docx-2-dhj-10.1177_20552076241271792 - Supplemental material for Developing, delivering, and evaluating an online course on socially assistive robots in culturally competent and compassionate healthcare: A sequential multiphase, mixed-method study Supplemental material, sj-docx-2-dhj-10.1177_20552076241271792 for Developing, delivering, and evaluating an online course on socially assistive robots in culturally competent and compassionate healthcare: A sequential multiphase, mixed-method study by Irena Papadopoulos and Runa Lazzarino in DIGITAL HEALTH
