# Supplementary material for: Developing, delivering, and evaluating an online course on socially assistive robots in culturally competent and compassionate healthcare: A sequential multiphase, mixed-method study
Source: Digit Health. 2024 Oct 29;10:20552076241271792. doi: 10.1177/20552076241271792 (PMC11528809; doi:10.1177/20552076241271792)
Supplement: sj-doc-1-dhj-10.1177_20552076241271792 - Supplemental material for Developing, delivering, and evaluating an online course on socially assistive robots in culturally competent and compassionate healthcare: A sequential multiphase, mixed-method study [file sj-doc-1-dhj-10.1177_20552076241271792.doc]

Supplementary material 1. MOOC Pre-course questionnaire

**About you**

**Your Name and Surname**

|  |
| --- |

**Your Email Address**

|  |
| --- |

**Your gender?**

|  |
| --- |

**Your country**

1. United Kingdom
2. Italy
3. Romania
4. Cyprus
5. Austria
6. Other, specify _________

**What is your qualification?**

1. Heath care professional
2. Social care professional
3. Teacher or trainer
4. Student/learner under qualification in heath or social care
5. Other, specify________

**What is your occupation?**

1. Working in health care institution
2. Working in social care institution
3. Working in education/training institution
4. Unemployed looking for a job
5. Student
6. Working in other sector­­­­­­­­­­­­­­, specify________

**On a scale of 1 to 5, what is your level of English?**

1. Poor
2. Fair
3. Good
4. Very good
5. Excellent

**On a scale of 1 to 5, rate your internet use skills**

1. Poor
2. Fair
3. Good
4. Very good
5. Excellent

**Did you take part in an online course in the past?**

Yes/Not

**Have you attended a course on the topic of Robots in heath or social care?**

Yes/Not

**What are the most important knowledge, skills and understandings you hope to increase in this course?**

- Knowledge on TRN specific issues in health and social care, education and practice
- Skills, linked to your professional profile
- Language skills;
- Digital competence and skills
- Cultural knowledge and skills of cultural communication;

**What other outcomes / benefits do you expect to achieve at the conclusion of the course? Please, make a list.**

|  |
| --- |

**How do you hope this course will benefit your professional work?**

|  |
| --- |

**Do you have any specific questions you would like the trainer to cover? Any other special requests?**

|  |
| --- |
