# Supplementary material for: Developing, delivering, and evaluating an online course on socially assistive robots in culturally competent and compassionate healthcare: A sequential multiphase, mixed-method study
Source: Digit Health. 2024 Oct 29;10:20552076241271792. doi: 10.1177/20552076241271792 (PMC11528809; doi:10.1177/20552076241271792)
Supplement: sj-docx-2-dhj-10.1177_20552076241271792 - Supplemental material for Developing, delivering, and evaluating an online course on socially assistive robots in culturally competent and compassionate healthcare: A sequential multiphase, mixed-method study [file sj-docx-2-dhj-10.1177_20552076241271792.docx]

Supplementary material 2. MOOC Post-course questionnaires

Questionnaire 1. Post-course Evaluation Questionnaire for MOOC Participants

**1. What are the most important knowledge, skills and understandings you have increased in this course**

- Awareness of some of the main reasons for SARs being used in health and social care settings
- Awareness of some of the main misconceptions and/or stereotypes that currently exist regarding the use of SARs in caring patients/clients
- Awareness of the cultural values, attitudes and views that health and social professionals may have about SARs
- Knowledge of different types of SARs and their various uses in health and social care
- Knowledge about the capabilities and the potential ‘role’ of SARs in health and social care
- Knowledge of some of the benefits and challenges related to usage of SARs in health and social care
- Understanding of the importance of communication between health and social care staff, the client and his/her family members, carers and SARs
- Knowledge about ethical and legal concerns associated with the safe implementation of SARs in health and social care
- Awareness of significance of collaborative teamwork between different stakeholders, including SARs and client/patient themselves, towards ensuring quality of patient/client care
- Understanding of how SARs can provide culturally sensitive and compassionate human-robot companionship to patients/clients in health and social care settings.
- Understanding about the practical knowledge and skills needed to work with SARs in health and social care
- Knowledge of potential issues related to physical and psychological safety of the patient/client when implementing SARs in health and social care

**2. How do you consider the course impacted you?**

- I improved the levels of skills, linked my professional profiles
- I have increased my knowledge on TRN specific issues in health and social care education and practice
- I have increased my language skills
- I have increased the level of my digital competence and skills for using different technologies
- I have increased cultural knowledge and skills of cultural communication
- I have a greater understanding and responsiveness to social, ethnic, linguistic and cultural diversity
- I have increased motivation and satisfaction in my work
- I have increased opportunities for employability and for professional development.

Questionnaire 2. IENE 10 MOOC Evaluation Questionnaire

**About you**

**Your Name and Surname**

|  |
| --- |

**Your Email Address**

|  |
| --- |

**Please, rate different statements, choosing True or False, as you agree or not agree with statements.**

1. The course met my training needs and my expectations
2. The course content was relevant and well structured
3. The course was well organized as timely, access to materials, level of the workload, etc.
4. The presentations where appropriate to my level of understanding, intuitive and friendly.
5. The learning tools and training materials were efficient for information and learning
6. There was a good balance of individual learning, group learning, practical and evaluation activities
7. The training activities were adequate facilitating my own learning pathway
8. The discussion forum was efficient for interaction with other trainees and stimulated my interest in the subject.
9. The instructors facilitated my learning, challenging and motivating me to do my best work and encouraging my to participate to group activities
10. I consider that I met my learning goals and objectives

**Please, rate the course overall.**

A. Poor

B. Fair

C. Good

D. Very good

E. Excellent
